# Effect of anti-glycosphingolipid monoclonal antibodies in pathogenic fungal growth and differentiation. Characterization of monoclonal antibody MEST-3 directed to Man*p*α1→3Man*p*α1→2IPC

**DOI:** 10.1186/1471-2180-10-47

**Published:** 2010-02-15

**Authors:** Marcos S Toledo, Loriane Tagliari, Erika Suzuki, Claudinei M Silva, Anita H Straus, Helio K Takahashi

**Affiliations:** 1 Division of Glycoconjugate Immunochemistry, Department of Biochemistry, Universidade Federal de São Paulo/Escola Paulista de Medicina, Ed. JL Prado, Rua Botucatu, 862, 04023-900, São Paulo, SP, Brazil; 2 Department of Microbiology, Immunology and Parasitology, Universidade Federal de São Paulo/Escola Paulista de Medicina, Rua Botucatu, 862, 04023-900, São Paulo, SP, Brazil

## Abstract

**Background:**

Studies carried out during the 1990's demonstrated the presence of fungal glycoinositol phosphorylceramides (GIPCs) with unique structures, some of them showed reactivity with sera of patients with histoplasmosis, paracoccidioidomycosis or aspergillosis. It was also observed that fungal GIPCs were able to inhibit T lymphocyte proliferation "in vitro", and studies regarding the importance of these molecules to fungal survival showed that many species of fungi are vulnerable to inhibitors of sphingolipid biosynthesis.

**Results:**

In this paper, we describe a detailed characterization of an IgG2a monoclonal antibody (mAb), termed MEST-3, directed to the *Paracoccidioides brasiliensis *glycolipid antigen Pb-2 (Man*p*α1→3Man*p*α1→2IPC). mAb MEST-3 also recognizes GIPCs bearing the same structure in other fungi. Studies performed on fungal cultures clearly showed the strong inhibitory activity of MEST-3 on differentiation and colony formation of *Paracoccidioides brasiliensis*, *Histoplasma capsulatum *and *Sporothrix schenckii*. Similar inhibitory results were observed when these fungi where incubated with a different mAb, which recognizes GIPCs bearing terminal residues of β-D-galactofuranose linked to mannose (mAb MEST-1). On the other hand, mAb MEST-2 specifically directed to fungal glucosylceramide (GlcCer) was able to promote only a weak inhibition on fungal differentiation and colony formation.

**Conclusions:**

These results strongly suggest that mAbs directed to specific glycosphingolipids are able to interfere on fungal growth and differentiation. Thus, studies on surface distribution of GIPCs in yeast and mycelium forms of fungi may yield valuable information regarding the relevance of glycosphingolipids in processes of fungal growth, morphological transition and infectivity.

## Background

Drouhet [1] described the existence of over 72,000 species of fungi widespread in nature, and more than 300 may be associated with human mycoses. In the last two decades, it was observed a dramatic raise in mortality of immunosupressed individuals associated with fungal infection. Although antifungal therapies have been successful and selective, the outbreaks of resistant strains, together with an increase on fungal tolerance levels to currently available antifungal, were described by several reports [1,2]. Therefore, a compelling search for novel antifungal therapies has been greatly stimulated. Studies carried out during the 1990s demonstrated that many species of fungi are vulnerable to inhibitors of enzymes of the sphingolipid biosynthesis pathway, such as inositol phosphorylceramide (IPC) synthase [3,4]. This particular enzyme transfers *myo*-inositol-1-phosphate from phosphatidylinositol to ceramide, the first and an essential step for the biosynthesis of glycoinositol phosphorylceramides (GIPCs), a class of complex anionic glycosphingolipids (GSLs) widely distributed among fungal species [5-7]. In this manner, GIPCs synthesis are highly susceptible to IPC synthase inhibitors, which in turn are remarkably toxic to many mycopathogens, but exhibit low toxicity in man, since the IPC or IPC-synthase gene are absent in mammals [5].

The detailed characterization of GIPCs from a variety of fungi revealed an extensive structural diversity. Based on further studies, more than 30 distinct GIPC structures have been identified to date, which may present one of the 3 well-confirmed core structures distinguishable at the monoglycosyl level and absent in mammals [5-7]. Some of these GIPCs have antigenic glycoside determinants, such as terminal β-D-galactofuranose residues, which are recognized by human sera, suggesting their potential as targets for immunodiagnostic and the possibility of therapy based on stimulation of mammalian humoral response [8-15]. It should be emphasized that the expression of these GIPCs is considerably dependent on species, and at least for some mycopathogens, strongly regulated during morphogenesis [8-11,13,16-23].

In this context, to investigate the role of GSLs in differentiation and colony formation of *Paracoccidioides brasiliensis*, *Histoplasma capsulatum*, and *Sporothrix schenckii*, we used three monoclonal antibodies (mAbs) raised to fungal GSLs: a) mAb MEST-1 directed to terminal Gal*f*β1→3/6Man*p *[13], b) mAb MEST-2 directed to β-glucosylceramide [24], and c) mAb MEST-3 directed to terminal Man*p*α1→3Man*p*α1→2Ins (this work). Table 1 summarizes the reactivity of mAbs MEST-1, -2 and -3: **i) **to lipids extracted from yeast and mycelium forms, which were analyzed by high performance thin layer chromatography (HPTLC) immunostaining, and **ii) **to yeast and mycelium forms of fungi used in this work, that were analyzed by indirect immunofluorescence (IFI). As shown in this paper, the availability of mAbs specifically directed to different GSL structures may be used as effective tools to a more accurate understanding of the organizational pattern and the biological role of GSLs of different fungi.

**Table 1:** Reactivity of mAbs MEST-1, -2 and -3, with different fungi preparation

		MEST-1 Gal*f*β1→3/6Man*p*		MEST-2 GlcCer		MEST-3 Man*p*α1→3Man*p*α1→2Ins	
				
		HPTLC	IFI	HPTLC	IFI	HPTLC	IFI
Pb	Y	**+**	**+**	**+**	**+**	**+**	**+**
	M	**+**	**-**	**+**	**-**	**+**	**-**
							
Ss	Y	**- **(np)	**- **(np)	**+**	**+**	**+**	**+**
	M	**- **(np)	**- **(np)	**+**	**-**	**- **(np)	**- **(np)
							
Hc	Y	**+**	**+**	**+**	**+**	**+**	**+**
	M	**- **(np)	**- **(np)	**+**	**-**	**- **(np)	**- **(np)

Reactivity of mAbs MEST-1, -2 and -3, with fungal glycolipids by HPTLC immunostaining (**HPTLC)**; and with fixed fungi by indirect immunofluorescence **(IFI). Pb **= *P. brasiliensis*; **Ss **= *S. schenckii*; **Hc **= *H. capsulatum*; **Y **= yeast; **M **= mycelium; **MEST-1 **recognizes epitope Gal*f*β1→3/6Man*p*; **MEST-2 **recognizes fungal glucosylceramide (GlcCer); **MEST-3 **recognizes epitope Man*p*α1→3Man*p*α1→2Ins; "**+**" indicates positive staining; "**-**" indicates negative staining, and "**np**" indicates epitope not present. [13,24].

## Results

### Characterization of mAb MEST-3

**Figure 1:**
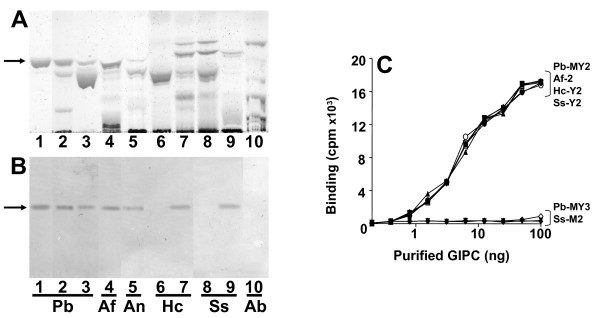
**Reactivity of fungal GIPCs with MEST-3**. Fungal GIPCs were purified by a combination of chromatography in DEAE-Sephadex, silica gel 60, HPLC and preparative HPTLC. HPTLC was developed in solvent A. **Panel A**, stained with orcinol/H_2_SO_4 _and **panel B**, immunostaining with MEST-3. Lane **1**, GIPC Pb-2 from mycelium form of *P. brasiliensis*; lane **2**, acidic GSLs from mycelium form of *P. brasiliensis*; lane **3**, acidic GSLs from yeast form of *P. brasiliensis *(Pb); lane **4**, acidic GSLs from hyphae of *A. fumigatus (Af)*; lane **5**, acidic GSLs from hyphae of *A. nidulans *(An); lane **6**, acidic GSLs from mycelium form of *H. capsulatum *(Hc); lane **7**, acidic GSLs from yeast form of *H. capsulatum*; lane **8**, acidic GSLs from mycelium form of *S. schenckii *(Sc); lane **9**, acidic GSLs from yeast form of *S. schenckii*; lane **10**, acidic GSLs from the edible mushroom *Agaricus blazei *(Ab). Arrows indicates chromatographic migration of Pb-2, Af-2, An-2, Hc-Y2 and Ss-Y2. Panel **C**, GIPCs (first well 0.1 μg) were serially double diluted in ethanol and adsorbed on a 96-well plate. MEST-3 (100 μl) was added and incubated overnight at 4°C. The amount of antibody bound to GSLs was determined by incubation with rabbit anti-mouse IgG (2 h) and 10^5 ^cpm of ^125^I-labeled protein A in 1% BSA. Pb-2 from yeast (closed square) and from mycelium (closed triangle) forms of *P. brasiliensis*; Ss-Y2 (open circle) from yeast form of *S. schenckii*; Af-2 (open triangle) from *A. fumigatus*, Hc-Y2 (open inverted triangle) from yeast forms of *H. capsulatum*, Pb-3 (closed inverted triangle) from yeast and Pb-3 (closed diamond) from mycelium forms of *P. brasiliensis *and Ss-M2 (open diamond) from mycelium forms of *S. schenckii*.

Aiming to study the biological role of GIPCs, and since expression of these glycoconjugates with terminal galactofuranose residues, which are recognized by MEST-1, is restricted to *P. brasiliensis *(Pb), *H. capsulatum *(Hc) and *A. fumigatus *(Af), we decided to develop a mAb directed to GIPC Pb-2, from *P. brasiliensis*, which structure Man*p*α1→3Man*p*α1→2IPC is expressed in a wide variety of fungi, and therefore a mAb directed to such structure would be highly desirable to detect a large number of pathogenic fungi. Among a few clones showing reactivity with GIPC Pb-2, a clone secreting an IgG2a monoclonal antibody was established, and termed MEST-3. By HPTLC-immunostaining (Figure 1B, lanes 1-3) it was observed that MEST-3 reacts with Pb-2 from yeast and mycelium forms of *P. brasiliensis*, and other GIPCs containing the same structure as Pb-2, such as Hc-Y2 from yeasts of *H. capsulatum *(Figure 1B, lane 7), Ss-Y2 from yeasts of *S. schenckii *(Figure 1B, lane 9), Af-2 from hyphae of *A. fumigatus *(Figure 1B, lane 4), and An-2 from hyphae of *A. nidulans *(Figure 1B, lane 5). Moreover, lanes 6 and 8 of Figure 1A-B confirm that mycelium forms of *H. capsulatum *and *S. schenckii *do not express GIPCs bearing the epitope recognized by MEST-3, as described before [8,9,22,23]. Also, by solid-phase radioimmunoassay (RIA), it was verified that mAb MEST-3 was able to detect as low as 5 ng of purified Pb-2, Hc-Y2, SS-Y2 and Af-2 (Figure 1C). Conversely, no reactivity of MEST-3 with GIPCs, presenting the structures Man*p*(α1→3) [Gal*f*(β1→6)]Man*p*(α1→2)IPC (Pb-3, Hc-Y3, Af-3); Manα1→2IPC (MIPC) and Manα1→3Manα1→6IPC (Ss-M2), was detected by HPTLC-immunostaining or RIA.

**Figure 2:**
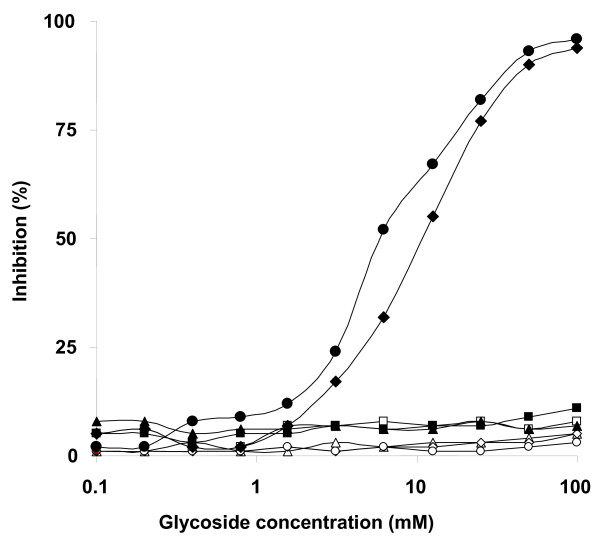
**Inhibition of mAb MEST-3 binding to Pb-2**. 96-well plates were adsorbed with GIPC Pb-2 from mycelium forms of *P. brasiliensis*. Methyl-glycosides, disaccharides and GIPC-derived glycosylinositols (first well 100 mM) were serially double diluted with PBS and preincubated with MEST-3, and the inhibition assay was carried out as described in *Materials and Methods*. The effects of the methyl-glycosides, disaccharides and glycosylinositols are expressed as percentages of inhibition of MEST-3 binding to Pb-2. (closed square) Man*p*α1→2Man*p*, (closed circle) Man*p*α1→3Man*p*, (closed triangle) Man*p*α1→6Man, (open diamond) methyl-α/β-D-glucopyranoside; (open circle) methyl-α/β-D-galactopyranoside; (open triangle) methyl-α/β-D-mannopyranoside, (closed diamond) Manα1→3Manα1→2Ins, (open square) Manα1→3Manα1→6Ins.

Treatment of Pb-2 with sodium *m*-periodate led to a decrease of 82% of mAb MEST-3 binding to this GIPC, indicating that MEST-3 recognizes the carbohydrate moiety of Pb-2 (data not shown), the structural features of the glycoepitope, recognized by MEST-3, was analyzed by inhibition assays on solid-phase RIA carried on 96-well plates pre-coated with purified Pb-2 antigen using different methyl-glycosides, disaccharides and glycosylinositols derived from GIPCs. As shown in Figure 2, methyl-α-D mannopyranoside, Manα1→2Man and Manα1→6Man did not inhibit MEST-3 binding to Pb-2, whereas disaccharide Manα1→3Man and glycosylinositol Manα1→3Manα1→2Ins, at a concentration of 25 mM, were able to inhibit by 80% the binding of MEST-3 to Pb-2 antigen. In addition, glycosylinositol Manα1→3Manα1→6Ins, derived from Ss-M2 of mycelium forms of *S. schenckii*, was not able to inhibit MEST-3 binding to Pb-2. Taking together, these data indicate that the epitope recognized by MEST-3 is not restricted to the terminal residue of mannose, but also includes the subterminal residues of mannose and *myo*-inositol (3mannoseα1→2*myo*-inositol). Therefore, these results clearly indicate that MEST-3 recognizes specifically GIPCs presenting the linear structure Man*p*α1→3Man*p*α1→2*myo*-inositol.

### Indirect immunofluorescence with MEST-3

**Figure 3:**
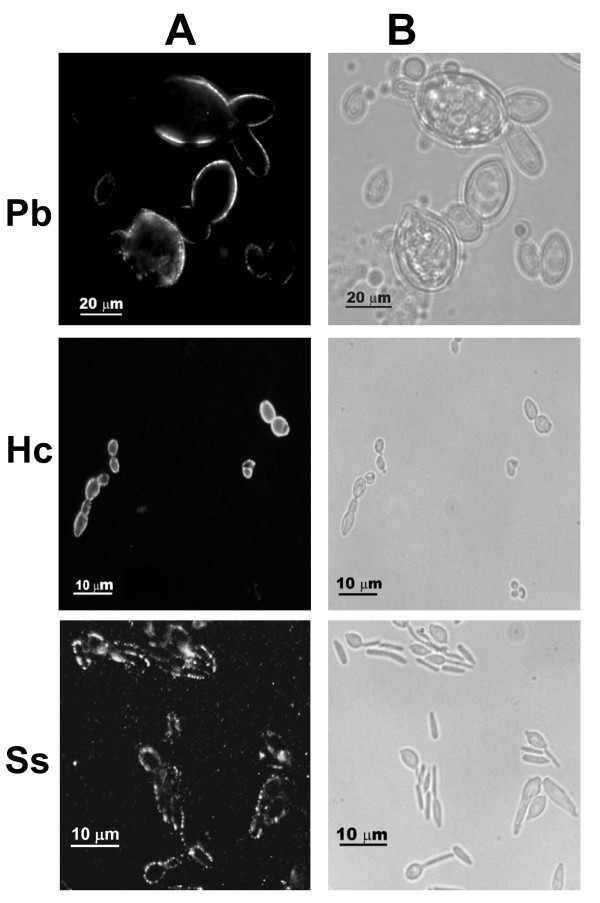
**Indirect immunofluorescence**. Indirect immunofluorescence of yeast forms of *P. brasiliensis *(Pb), *H capsulatum *(Hc) and *S. schenckii *(Ss), with mAb MEST-3. **A**- fluorescence. **B**- phase contrast.

As shown in Figure 3, indirect immunofluorescence using MEST-3 showed that yeast forms of *P. brasiliensis *and *H. capsulatum *present homogenous surface labeling, whereas yeast forms of *S. schenckii *present an interspersed labeling at cell surface. By HPTLC immunostaining or RIA, mAb MEST-3 showed reactivity with GIPCs isolated from mycelium forms of *P. brasiliensis *and hyphae of *A. fumigatus *and *A. nidulans *(Figure 1A-C), but it is noteworthy that no fluorescence was observed with mycelium forms of *P. brasiliensis *and hyphae of *A. fumigatus *and *A. nidulans *(not shown). As expected, by immunostaining and RIA (Figures 1A-C), no reactivity of MEST-3 was observed with mycelium forms of *S. schenckii *and *H. capsulatum*. Negative controls using an irrelevant mAb showed no fluorescence (not shown).

### Effect of monoclonal antibodies on fungal growth

**Figure 4:**
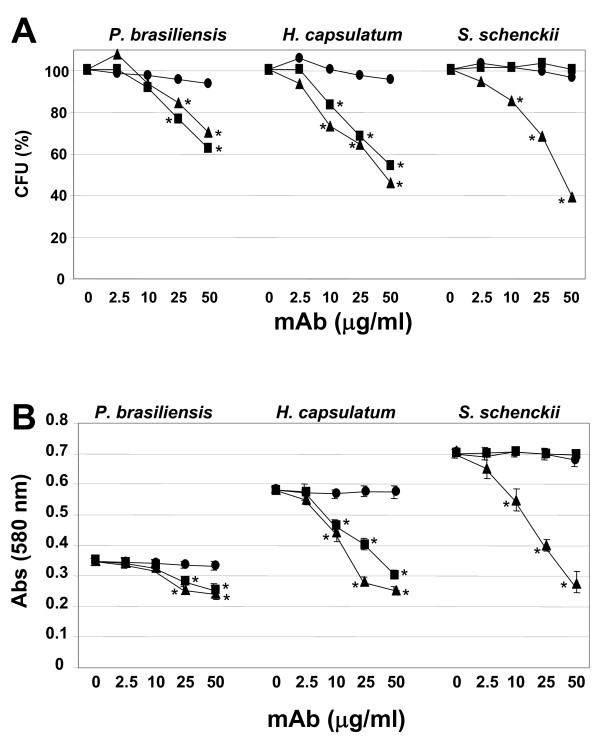
**Effect of monoclonal antibodies on fungal growth**. **Panel A**, Yeast forms of *P. brasiliensis*, *H. capsulatum *and *S. schenckii *were incubated for 24 h with mAbs, or a control IgG or left alone, at 37°C. Yeasts were transferred to a petri dish containing PGY or BHI-agar medium, and incubated for 2 days at 37°C. Colony forming units (CFUs) were counted, and expressed as percentage of those incubated with an irrelevant mAb, considered as 100% of CFU. **Panel B**, MTT assay of fungi after incubation with mAbs MEST-1, -2, and -3. Yeast forms of *P. brasiliensis*, *H. capsulatum *and *S. schenckii *were incubated with mAbs, a control IgG or left alone. After incubation for 48 h, MTT solution was added to each well and was further incubated for 3 h at 37°C. The absorption was measured at 580 nm. MEST-1 (closed square), MEST-2 (closed circle) and MEST-3 (closed triangle). * p < 0.05.

By counting the total number of colony forming units (CFUs), the effect of mAbs MEST-1, -2 and -3 at different concentrations on fungal growth was analyzed. Under the conditions described in Methods, it was determined for *P. brasiliensis*, *H. capsulatum *and *S. schenckii*, a total of 57 ± 4, 41 ± 3 and 79 ± 4 CFUs, respectively. As shown in Figure 4A, mAbs MEST-1 and -3 were effective in inhibiting *P. brasiliensis *and *H. capsulatum *CFUs in a dose-dependent manner. mAb MEST-1 was able to inhibit *P. brasiliensis *and *H. capsulatum *CFU by about 38% and 45%, respectively, while MEST-3 inhibited *P. brasiliensis*, *H. capsulatum *and *S. schenckii *CFUs by about 30%, 55% and 65%, respectively (*p < 0.05). Conversely, as expected, MEST-1 was not able to inhibit *S. schenckii *CFU, since this fungus does not present glycolipids containing terminal residues of β-D-galactofuranose [22,23]. It should be noted that MEST-2 did not present significant CFU inhibitory activity in none of the three fungi used in this study. Confirming these results, *P. brasiliensis*, *H. capsulatum *and *S. schenckii *were grown in media containing mAbs for 48 h, after that, MTT {3-(4,5-Dimethylthiazol-2-yl)-2,5-diphenyltetrazolium bromide} was added to measure the growth rate. As observed in Figure 4B, MEST-1 and -3 inhibited significantly the growth of *P. brasiliensis *and *H. capsulatum*, whereas for *S. schenckii*, only MEST-3 was able to inhibit fungal growth.

### Effect of monoclonal antibodies on fungal dimorphism

**Figure 5:**
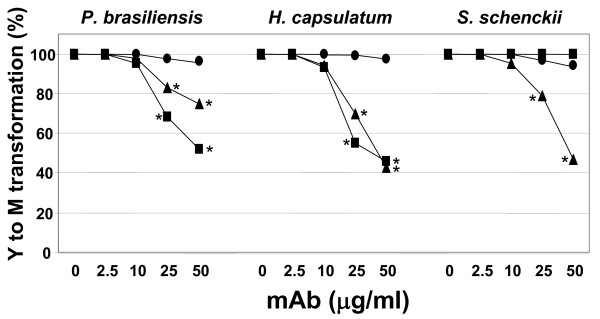
**Effect of monoclonal antibodies on yeast to mycelium transformation**. Yeast forms of *P. brasiliensis*, *H. capsulatum *and *S. schenckii *were incubated for one hour with different concentration of MEST-1, -2 and -3, and control IgG at 37°C. After that the yeast cultures were transferred to a 25°C incubator, and kept for 2 days. Three hundred yeasts were counted, and the number of yeast showing hyphae growth was evaluated. In control experiment 100% of yeast showed hyphae formation; the results represent the percentage of those incubated with an irrelevant mAb, considered as 100% of yeast to mycelium transformation. MEST-1 (closed square), MEST-2 (closed circle) and MEST-3 (closed triangle). * p < 0.05.

In order to analyze the effect of mAbs MEST-1, -2 and -3 on yeast to mycelium transformation of *P. brasiliensis*, *H. capsulatum *and *S. schenckii*, at first, yeast forms were incubated with these mAbs for 48 h at 25°C, which is the optimum temperature for mycelia growth. As observed for CFU, mAbs MEST-1 and -3 were also able to inhibit in a dose-dependent manner the yeast to mycelia differentiation of *P. brasiliensis *and *H. capsulatum *(Figure 5). In these experiments, 50 μg/ml of MEST-1 was able to inhibit the conversion of approximately 50% of *P. brasiliensis*, and 55% of *H. capsulatum *from yeast to mycelia. Under the same condition, MEST-1 was not able to inhibit the conversion from yeast to mycelia in *S. schenckii *(Figures 5). Moreover, mAb MEST-3 was able to inhibit the conversion of yeast to mycelia of approximately 30% of *P. brasiliensis*, 55% for *H. capsulatum *and 50% for *S. schenckii *(Figure 5).

**Figure 6:**
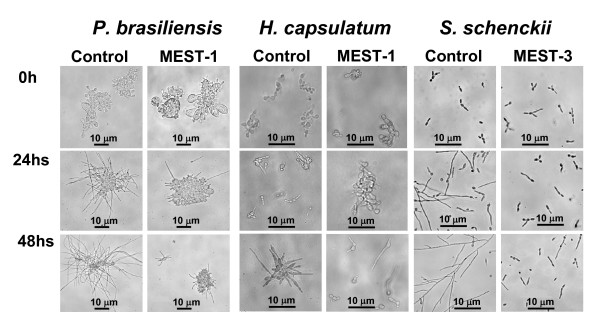
**Effect of mAbs on mycelia formation**. Yeasts were suspended in 1 ml of PGY or BHI medium. This suspension was added to a 24-well plate and supplemented with mAb MEST-1 or -3 (50 μg/ml), after one hour at 37°C cells were placed at 25°C. After 24 and 48 h of incubation yeast differentiation into mycelia forms was observed in an inverted microscope. Controls experiments were performed identically in presence of irrelevant immunoglobulins (normal mouse total Ig).

Furthermore, considering the relative proportion of yeast and mycelia forms as well the hyphal length, it was verified that mAb MEST-1 (Figure 6) and MEST-3 (not shown) were able to inhibit *P. brasiliensis *and *H. capsulatum *yeast to mycelia differentiation as early as 24 h after mAb incubation. Additionally, only MEST-3 (Figure 6) was able to inhibit *S. schenckii *yeast to mycelium differentiation. In contrast, no inhibition of yeast to mycelium differentiation was observed upon incubation of these fungal species with MEST-2 (Figure 5). Parallel experiments showed that after washing and replacing medium containing mAbs by antibody-free medium; the fungi tested were able to restore their growth and/or transformation, indicating that mAbs MEST-1, -2 and -3 present a fungistatic effect (data not shown).

**Figure 7:**
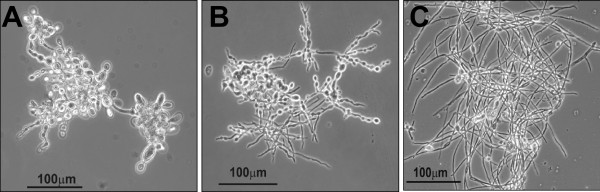
**Effect of mAb MEST-3 on yeast formation**. *P. brasiliensis *hyphae fragments were suspended in 1 ml of PGY medium and supplemented or not with mAb MEST-3 (50 μg/ml). Cells were placed on a 24-well plate at 37°C, and after 96 h of incubation, hyphae differentiation into yeast (M→Y) forms was observed under microscope. **Panel A **shows M→Y differentiation in free-mAb medium, **Panel B **shows M→Y differentiation in medium containing MEST-3, and **Panel C **shows the mycelia growth of hyphae fragments maintained at 23°C for 96 h in free-mAb medium.

In another set of experiments, it was evaluated the effect of mAb MEST-1 and -3 on *P. brasiliensis *mycelium to yeast transformation*s*, and as expected, it was not observed a significant inhibition, since these antibodies do not react or react weakly with mycelium forms. Thus, 50 μg/ml of MEST-1 and MEST-3 inhibited, respectively, 6% and 9% the transition from mycelium to yeast of *P. brasiliensis*. Figure 7 shows the differentiation of *P. brasiliensis *mycelia forms grown in presence (Panel B) or not (Panel A) of MEST-3 for 48 h at 37°C. In order to illustrate the differentiation inhibition, but not picturing the real inhibition percentage, Figure 7B pictured a field with high concentration of hyphae form.

## Discussion

### mAb MEST-3 specificity

In this paper, we describe the characterization of MEST-3, an IgG2a monoclonal antibody directed to the structure (Man*p*α1→3Man*p*α1→2IPC) from GIPC Pb-2 of *P. brasiliensis*. Among different methyl-glycosides, disaccharides and glycosylinositols, only Man*p*α1→3Man*p *and Man*p*α1→3Man*p*α1→2Ins inhibited MEST-3 binding to Pb-2 in solid-phase RIA. Furthermore, MEST-3 was unable to recognize, by solid-phase RIA or HPTLC-immunostaining, the intact GIPC Ss-M2 (Man*p*α1→3Man*p*α1→6IPC), thus suggesting that α1→6 linkage of the subterminal mannose unit to inositol represents a sterical hindrance for antigen recognition by MEST-3. Therefore, the minimum epitope required for optimum binding of MEST-3 to Pb-2 and similar GIPCs, would comprise the two linear mannose residues in specific linkage and the *myo*-inositol residue as follows: Man*p*α1→3Man*p*α1→2Ins.

By indirect immunofluorescence, it was observed that MEST-3 is reactive only with yeast forms of *P. brasiliensis*, *H. capsulatum *and *S. schenckii*, which is in agreement with previous data describing the crypticity of GIPC Pb-3 and GlcCer in mycelium forms of *P. brasiliensis *[13,24]. Accordingly, despite the detection of the GIPC Pb-2 extracted from hyphae of *P. brasiliensis *by HPTLC and HPTLC immunostaining with mAb MEST-3, it should be noted the complete lack of MEST-3 reactivity by immunofluorescence with fixed mycelia forms. Taking together, these data suggest that a particular organization of GSLs and/or cell wall structure, distinct from that found in yeast forms, is required for mAb MEST-3 binding to fungal GIPC Pb-2. In this context, the access of antibodies directed to GSLs of mycelium forms seems to be strongly affected by organizational or structural aspects that do not favor the interaction antigen-antibody.

### Growth and dimorphism inhibition by anti-glycosphingolipid mAbs

There are several reports in the literature showing the importance of neutral glycosphingolipids, such as cerebrosides, on fungal growth and morphological transition [25-27]. Rodrigues *et al*. [28] described that the addition of purified human antibodies, directed to GlcCer from *Cryptococcus neoformans*, inhibited cell budding and growth of this fungus. Therefore, the effects of three mAbs (MEST-1, -2 and -3), directed to different fungal GSLs, were analyzed on colony formation (CFU) of pathogenic dimorphic fungi (*P. brasiliensis*, *H. capsulatum *and *S. schenckii*). Experiments using mAb MEST-2, directed to fungal GlcCer, showed no significant inhibition of CFU or effect in dimorphism of the fungi studied. These data do not corroborate the results from Rodrigues *et al. *[28]. Possible explanations for these results may be related to the source of the antibodies, human and murine, in our case, or fungal species, since this effect was only observed in *C. neoformans*.

Our results using mAb MEST-1, directed to Pb-3 and Hc-Y3, showed significant inhibition of fungal growth and differentiation of *P. brasiliensis *and *H. capsulatum *from yeast to mycelia. As expected, no inhibition with MEST-1 was observed for *S. schenckii*, since this specie does not express galactofuranose-bearing GSLs. On the other hand, MEST-3 was able to inhibit CFU, fungal growth and differentiation of all three fungi studied. MEST-3 was able to cause higher inhibition of CFU and differentiation for *H. capsulatum *and *S. schenckii *than for *P. brasiliensis*. This lower degree of inhibition showed by *P. brasiliensis *could be attributed to the low GIPC Pb-2 concentration in yeast forms of this fungus [10]. On the other hand, GIPCs Hc-Y2 and Ss-Y2, which bear the same structure as Pb-2, represent about 30% and 20% of acidic glycolipid fraction from *H. capsulatum *and *S. schenckii *yeast forms respectively [8,23].

Conversely, results observed in the mycelium to yeast transformation, were not straightforward, a possible explanation could be related to the non-reactivity of mAbs MEST-1, -2 and -3, with mycelia forms, as observed by immunofluorescence assay (Table 1). Moreover, in *H. capsulatum *and *S. schenckii*, the transformation of mycelium to yeast takes at least three weeks in normal conditions, and the mycelium web hinders clear yeast observation and quantification.

It is now well established that the precise build up of lipid rafts is necessary to efficiently guide signal transduction through cell membrane [29], some new evidences indicate that in fungi, these constructions are also necessary for fungal survival and maintenance of the infection [30]. Two attractive hypotheses for the inhibitory activity of mAbs MEST-1 and MEST-3 on the yeast growth and differentiation would be: **i) **the association mAb-GIPCs sterically inhibits the formation of functional glycosphingolipid-enriched microdomains (GEM), thus blocking the action of key enzymes responsible for cell wall synthesis [31], and **ii) **an already existing microdomain to which a mAb-GIPC association takes place is not functional, hindering essential signal transduction to fungal growth and/or dimorphism. Confocal microscopy, with *P. brasiliensis *cell wall stained with calcofluor white indicates two places of MEST-3 binding, **i) **extensive and internal to calcofluor labeling, i.e. plasma membrane, and **ii) **discrete external as well as co-labeling with calcofluor, these preliminary data led us to suggest a new conceptual model of GSL arrangements in yeast forms where microdomain-like regions containing GIPCs could also be located at the external surface of the cell wall. Further work to substantiate this concept model is under investigation in our laboratory aiming an extensive comprehension of fungal glycosphingolipid enriched microdomains regarding their composition, surface localization, role in signaling processes and possible role in host cell binding and infection.

This study and others have shown that specific GIPCs are found in a large variety of pathogenic fungi [6,7]. In some cases, those GIPCs are recognized by sera from patients with paracoccidioidomycosis, histoplasmosis or aspergillosis [8-10,13-15,32], indicating that GIPCs are immunogenic and able to induce the production of human antibodies during fungal infections. The broad distribution of GIPCs in pathogenic fungi and the antifungal activity of monoclonal antibodies directed to GIPCs indicate that these molecules may represent potential targets for the development of new therapeutical approaches based on induction of protective antibodies.

## Conclusion

The fine specificity of MEST-3 was assessed by inhibition assays using different methyl-glycosides, disaccharides and oligosaccharides. Only Manα1→3Man and the glycoinositol Manα1→3Manα1→2Ins, from Pb-2, were able to inhibit, by about 95%, MEST-3 binding to Pb-2 antigen of *P. brasiliensis*. The epitope recognized by MEST-3 was defined as Manα1→3Manα1→2Ins; this structure was already described in a variety of pathogenic fungi [5-11,15-17,19,23]. Studies using mAbs MEST-3 and MEST-1, as fungal growth inhibitors showed that anti-GIPCs mAbs presented a strong inhibitory activity on growth, differentiation and colony formation of *P. brasiliensis*, *H. capsulatum*, and *S. schenckii*. On the other hand, no statistically significant inhibition was observed with anti-GlcCer (MEST-2).

These results strongly suggest that mAbs directed to particular glycosphingolipids are able to interfere on fungal growth and differentiation. An attractive hypothesis is that, by hindering the formation of functional lipid rafts by association with mAbs, a possible dependence of surface organization of glycosphingolipids in fungi is essential for different fungal processes, such as growth, morphological transition, and infectivity.

## Methods

### Fungal isolates and growth conditions

*Paracoccidioides brasiliensis *strain Pb18 was provided by Dr Z.P. Camargo, São Paulo, SP, Brazil. Yeast and mycelia forms of *P. brasiliensis *were grown at 37°C and 25°C, respectively, in PGY (peptone 5 g/L, glucose 15 g/L, yeast extract 5 g/L) using 2.5 L Fernbach flasks in a shaker at 100 rpm [10]. *Histoplasma capsulatum *strain 496 from human pulmonary lesion [33] and *Sporothrix schenckii *strain 65 from human foot cutaneous lesion [22,23], were kindly provided by Dr O. Gompertz, São Paulo, SP, Brazil. Yeast and mycelia forms of both fungi were grown in Brain Heart Infusion (BHI) (37 g/L) at 37°C and 25°C, respectively. After 5-7 days both yeast and mycelia forms of the various fungi were inactivated with 0.1% of thimerosal, and after an additional 48 h the fungi were collected by filtration on Whatman n° 1 filter paper, except for yeast forms of *S. schenckii *and *H. capsulatum*, which were harvested by centrifugation at 5,200 × g for 20 minutes.

### Extraction and purification of glycosphingolipids (GSLs)

GSLs were extracted by homogenizing yeast or mycelia forms (~ 30 g) in an Omni-mixer (Sorvall Inc. Wilmington, DE), three times with 200 ml of isopropanol/hexane/water (IHW, 55:20:25, v/v/v, upper phase discarded), and twice with 200 ml of chloroform/methanol (CM, 2:1, v/v). The five extracts were pooled, dried on rotary evaporator, dialyzed against water and lyophilized. Neutral and acidic GSLs were separated in a DEAE-Sephadex A-25 column as described by Yu and Ledeen [34]. Fractions containing GIPCs, were assessed by HPTLC on silica gel 60 plates (E. Merck, Darmstadt, Germany) using solvent A: chloroform/methanol/CaCl_2 _0.02%, (60:40:9; v/v/v), and stained with orcinol/H_2_SO_4_. For preparative-scale HPTLC separated GSL bands were visualized under UV light after spraying with primulin 0.01% in 80% aqueous acetone [35]. GSLs were isolated from silica gel scraped from the plates by repeated sonication in IHW, as described [36].

### Production of hybridomas

About 600 μg of GIPC Pb-2 purified from mycelia forms of *P. brasiliensis *were dissolved in 1.5 ml of distilled water and mixed with 1.5 mg of acid-treated heat-inactivated *Salmonella minnesota*. Aliquots (100 μl) of this suspension containing 40 μg of the antigen were used to immunize six weeks old BALB/c mice, by i.v. route, through the caudal vein once a week, over 4 weeks. After a rest period of 30 days, the immune response was boosted with 200 μl of the immunogenic complex. Three days later, the mice were sacrificed and their spleen removed. The lymphocytes were fused with NS-1 myeloma cells and placed in 96-well plates. Solid-phase RIA detected hybrids secreting immunoglobulins reacting with Pb-2. Only clones showing strong reactivity with Pb-2 of mycelia and yeast forms of *P. brasiliensis *were cloned by limited dilution as described [13,24,37]. Research ethical approval (CEP 0023/06) was granted by the Ethical Research Committee Boards of Universidade Federal de São Paulo.

### Binding assay

Various GSLs were adsorbed on 96-well plates (Falcon Microtest III flexible assay plates, Oxnard, CA). Solutions (25 μl/well, 100 ng/first well) in ethanol of different GSLs were serially diluted, dried at 37°C and wells blocked with 1% bovine serum albumin (BSA) in 0.01 M phosphate-buffered saline (PBS), pH 7.2 (200 μl) for 2 h, and sequentially incubated with mAb MEST-3 (100 μl) overnight at 4°C, rabbit anti-mouse IgG (50 μl) for 2 h, and with 50 μl of ^125^I-labeled protein A in PBS with 1% of BSA (about 10^5 ^cpm/well) for 1 h. The amount of mAb MEST-3 bound to Pb-2 was determined by measuring the radioactivity in each well in a gamma counter [13].

### Release of glycosylinositols by ammonolysis

Ammonolysis of GIPCs was performed as described by Barr and Lester [8] and Levery *et al. *[11]. Briefly, 100 μg of GIPCs Pb-2 and Ss-Y2 were heated in a Teflon-lined screw-capped test tube with 10 N NH_3_.H_2_0 (~ 1 mL) for 18 h at 150°C. The solution was cooled and evaporated under N_2 _stream at 37°C; this process was repeated after addition of a few drops of 2-propanol. The residue was sonicated in 1 mL of water and the lipophilic components were removed by passage of this solution through a small C18-silica solid-phase extraction cartridge, washing twice with 1 ml of water. The combined aqueous fraction containing free glycosylinositol was lyophilized and used for inhibition of antibody binding to GIPCs Pb-2.

### Inhibition of antibody binding by different methyl glycosides, disaccharides and glycosylinositols

Initially, 75 μl of a 200 mM solution of several methyl-α- and β-D-glycosides (glucopyranoside, galactopyranoside and mannopyranoside), disaccharides (Manα1→2Man, Manα1→3Man and Manα1→6Man), purchased from Sigma (MO, USA), and the glycosylinositols (Manα1→3Manα1→2Ins and Manα1→3Manα1→6Ins, described above), were serially diluted with PBS in a 96-well plate. Each glycoside solution was incubated with 75 μl of MEST-3 at room temperature [35]. After 2 h, aliquots of 100 μl were taken and incubated overnight at 4°C in 96-well plates pre-coated with the GIPC Pb-2 (100 ng/well) essentially as described under Binding assay.

### Periodate oxidation

Ninety-six-well plates were coated with different concentrations (100 ng to 5 pg) of GIPC Pb-2 and treated with 5 and 20 mM of sodium *m*-periodate in PBS (0.1 M, pH 7.0) at room temperature for 30 min [13]. The plates were washed with PBS, reduced with NaBH_4 _(50 mM in PBS) during 30 min, blocked with 5% of BSA in PBS for 1 h, and incubated overnight with mAb MEST-3, and processed as described in Binding Assay.

### High performance thin layer chromatography (HPTLC) immunostaining

GIPCs purified from different fungi were separated by HPTLC, and the immunostaining of the plates was performed by the procedure of Magnani *et al. *[38], modified by Zuolo *et al*. [39] and Takahashi *et al*. [40]. GSLs (3 μg) were separated in solvent A: C:M:CaCl_2 _0.02% (60:40:9; v/v/v), after development, the plates were dried, soaked in 0.5% polymethacrylate in hexane, dried, and blocked for 2 h with 1% of BSA in PBS. Plates were then incubated with mAb MEST-3 overnight followed by sequential incubations with rabbit anti-mouse IgG and ^125^I-labeled protein A (2 × 10^7 ^cpm/50 ml of BSA/PBS).

### Indirect immunofluorescence

Fungi were fixed with 1% formaldehyde in PBS for 10 min. Cells were washed, suspended in 1 ml of PBS, and 20 μl of the solution was added to a coverslip pre-treated with poly-L-lysine 0.1% during 1 h. Air dried preparations were soaked for 1 h in PBS containing 5% of BSA, and incubated subsequently with culture supernatant of mAb MEST-3 (2 h), biotin-conjugated goat anti-mouse IgG (1 h), and with avidin-conjugated fluorescein (1 h). After each incubation the coverslips were washed five times with PBS. The coverslips were examined with an epifluorescence microscope [13]. Control experiments for each fungus were carried out, in the presence of an irrelevant monoclonal antibody, and no fluorescence was observed.

### Cell growth

To evaluate the influence of mAbs directed to GSLs on the growth of different fungi, yeasts (10^4^/ml) were incubated in 96-well plate in the presence of mAbs MEST-1, -2, or -3 for 24 h at 37°C, in concentration ranging from 2.5 to 50 μg/ml. The growth rate was evaluated by two procedures; **1) **viable CFU were evaluated by plating 100 μl of the samples onto BHI or PGY agar plates, followed by incubation for 2 days at 37°C, and colony counting; or **2) **5 μl of 3-(4,5-Dimethylthiazol-2-yl)-2,5-diphenyltetrazolium bromide (MTT) solution (5 mg/ml MTT in phosphate-buffered saline, pH 7,4) were added to each well and the plates were further incubated at 37°C, for 3 h, after incubation the medium containing MTT was partially removed, and dimethyl sulfoxide (100 μl) was added to solubilize the MTT formazan product [41]. The absorbance of each well was measured at 580 nm by a microtiter ELISA plate reader. Control systems were similarly treated with an irrelevant immunoglobulin (normal mouse total Ig) and plated. All experiments were repeated three times in triplicates, and the results shown are a representative of these experiments.

### Fungal differentiation - yeast to mycelium

10^4 ^viable yeasts were suspended in 1 ml of PGY (*P. brasiliensis*) or BHI (*H. capsulatum *and *S. schenckii*) medium. The suspension was incubated in a 24-well plate and supplemented with mAb MEST-1, -2, or -3 (at a concentration of 2.5, 10, 25 or 50 μg/ml), after one hour at 37°C, 24-well plate was transferred to a 24°C incubator and kept for 2 days. The number of yeast showing hyphae growth was counted, and presented as percentage of those incubated with irrelevant immunoglobulins (normal mouse total Ig). In control experiment 100% of yeast showed hyphae formation. For each mAb concentration, 300 viable fungi (yeast or transforming-yeast) were counted, yeasts differentiating into mycelia forms were observed and evaluated considering hyphae formation. Control experiments were performed identically, with the addition of irrelevant immunoglobulins. Experiments were performed in triplicate sets and representative results are shown in Figure 5.

### Fungal differentiation - mycelium to yeast

A 5 days old culture containing hyphae, was washed and combined in a tube with sterile PBS and 5 mm glass beads, this suspension was agitated in vortex (3 × 5 min), to broke the web mycelia in small hyphae. After decantation, the supernatant containing short lengths of hyphae was centrifuged and the hyphae suspended in 1 ml of PGY medium. The suspension was incubated in a 24-well plate and supplemented with mAb MEST-1, -2, or -3 (at a concentration of 2.5, 10, 25 or 50 μg/ml), at 37°C. After 48 h and 96 h of incubation cultures were analyzed under inverted microscopy. Controls experiments were performed identically, with the substitution of mAb to irrelevant immunoglobulins (normal mouse total Ig).

## Authors' contributions

MST, AHS and HKT planned, designed the study, and wrote the main draft of the paper. MST produced the mAb, developed the experiments, the data analysis and prepared the figures. ES developed experiments, supports the discussion of the results and revised the manuscript. LT and CMS performed microscopy experiments. All authors have read and approved the final manuscript.
